# Experiences and Challenges of Patients With Chronic Respiratory Disease During a Virtual Research Study: Qualitative Study

**DOI:** 10.2196/80216

**Published:** 2026-07-09

**Authors:** Noah Tregobov, Austin McMillan, Aditya Dhariwal, Y Sarah Chae, Bahareh Poureslami, Maryam Mahjoob, Joshua Block, Iraj Poureslami

**Affiliations:** 1 Vancouver—Fraser Medical Program, The University of British Columbia, 317-2194 Health Sciences Mall, Vancouver, BC, V6T 1Z3, Canada, 1 604 822 2421; 2 Division of Respiratory Medicine, Centre for Heart and Lung Health, Vancouver Coastal Health Research Institute, Vancouver, BC, Canada; 3 Schulich School of Medicine & Dentistry, Western University, London, ON, Canada; 4 Department of Occupational Science and Occupational Therapy, The University of British Columbia, Vancouver, BC, Canada; 5 Centre for Aging SMART Rehabilitation Research Program, Vancouver Coastal Health Research Institute, Vancouver, BC, Canada; 6 Faculty of Health Sciences, Simon Fraser University, Vancouver, BC, Canada; 7 Cancer Control Research, BC Cancer Agency, Vancouver, BC, Canada; 8 Bienen School of Music, Northwestern University, Evanston, IL, United States; 9 Deparment of Mechanical and Materials Engineering, Queen's University, Kingston, ON, Canada

**Keywords:** virtual research, respiratory diseases, qualitative methods, research challenges, health literacy

## Abstract

**Background:**

Virtual research has emerged as a promising and convenient approach. This study investigates the experiences and challenges faced by patients with chronic respiratory diseases who participated in a virtual research study.

**Objective:**

This study aimed to explore the experiences of adults with chronic respiratory diseases participating in a virtual research study and to identify challenges they encountered to inform future virtual research design and implementation.

**Methods:**

Adults with a diagnosis of asthma or chronic obstructive pulmonary disease taking medications for their condition were recruited from the Greater Vancouver Area. A total of 185 participants completed an asthma-specific or chronic obstructive pulmonary disease–specific health literacy measurement tool through telephone interviews and then completed a postinterview checklist (n=36) or survey (n=149) on their experience with virtual research. To test the health literacy tool’s reproducibility, 110 participants were retested and completed the same checklist (n=79) or survey (n=31). Interviews were transcribed and thematic analyses were conducted on interviews, surveys, and checklists.

**Results:**

Six overarching themes were identified: overall experience with virtual research, clarity of instructions, technology, communication, condition and cognition, and logistics. While participants generally reported a positive experience, the study identified significant challenges, including technological barriers, the need for clear and concise instructions, mental fatigue, and communication difficulties.

**Conclusions:**

These findings highlight the importance of providing comprehensive technical support, ensuring clear communication, and addressing ethical considerations in virtual research. This study contributes valuable insights to the growing body of literature on the viability and challenges of virtual research in health care.

## Introduction

The COVID-19 pandemic disrupted research and health care delivery, resulting in transitions to virtual platforms [1,2]. Health care professionals increasingly used eHealth tools, defined as the provision of health care services to patients through the use of technology [3]. Additionally, researchers, particularly qualitative researchers, have applied virtual data collection methods such as online surveys and virtual interviewing through video and telephone calls, emails, and SMS text messages [4]. These methods offer the benefits of synchronous or asynchronous communication [5]. The various approaches of virtual research have unique advantages and disadvantages for both researchers and participants.

Advantages of virtual research include cost and time efficiency, flexible scheduling, and more representative samples by reducing barriers to travel, enabling geographically dispersed and mobility-friendly participation [6-8]. Additionally, virtual methods often provide a higher degree of perceived and actual anonymity, which may encourage participation from vulnerable groups and facilitate the discussion of sensitive topics [5,6,8,9]. Consequently, studies show that virtual data collection methods can be as effective as traditional in-person approaches [10-17].

However, virtual research presents challenges such as technological barriers, lessened data familiarity, and shortened participant engagement. Researchers have limited control over participants’ technological issues such as internet connectivity, device compatibility, and familiarity with technology [4,18]. Additionally, sampling bias may occur, favoring participants with better technological skills [18,19]. Advances in technology have led to the utilization of automatic transcription in videoconferencing software; however, this may lead to researchers becoming less familiar with their data compared with manual transcription [4]. Both participants and researchers may also be susceptible to “Zoom fatigue” or distractions at home, such as interruptions from children and pets, and on-screen notifications leading to reduced engagement [4,20].

Privacy and data integrity concerns represent critical challenges to both the participant and the researcher in virtual research, especially when using international technology platforms. Platforms such as Zoom (Zoom Communications, Inc) and Microsoft Teams (Microsoft Corporation) offer Health Insurance Portability and Accountability Act–compliant versions with encryption, but they are potentially vulnerable to data breaches, cyberattacks, and third-party data-handling practices [21-24]. In Canada, the regulatory landscape governing health data privacy in research is complex, characterized by overlapping federal and provincial privacy laws without harmonized national standards. While not a specific aim of this research paper, it is important to follow Canadian privacy legislation which requires safeguards proportionate to data sensitivity. International data sharing is permitted, but researchers remain accountable for protecting participant privacy which is relevant when using cloud-based platforms hosted outside Canada [25-28]. Researchers should also be aware of the potential of fraudulent participants, who may have ulterior motives for participating in research, and consider incorporating ways to minimize and detect fraudulent participation [29-34].

While several studies discuss potential challenges and offer recommendations for virtual research, these are mostly commentaries focusing on researchers’ perspectives [5,35]. The lived experiences and challenges of participants, especially within specific demographic cohorts such as patients with chronic respiratory disease (CRD), are important. To address this gap and identify areas to improve participant experience and study quality, this paper explores the experiences of patient participants with CRD in a virtual research study using qualitative methods. It is part of a larger study validating a health literacy (HL) tool for patients with CRD through virtual or phone interviews [36]. The primary objective of this substudy was to understand the experiences of patients with CRD with virtual research methods participating in the HL measurement tool study.

## Methods

### Ethical Considerations

The study received approval from The University of British Columbia Office of Behavioral Research Ethics (H15-01954).

### Participant Recruitment and Eligibility

Participants were recruited between August 2019 and June 2020. A research recruitment service was used where community patients were reached via random phone callouts in the Greater Vancouver Area. Patients were offered to receive a call back if they were interested in the study and met the eligibility criteria. Eligibility criteria included the following: (1) aged 19 years and older, (2) able to read and communicate in English, (3) physician-diagnosed asthma and/or chronic obstructive pulmonary disease, and (4) current use of a medication for asthma and/or chronic obstructive pulmonary disease.

### Survey and Checklist Development

The study involved 2 stages: first, a 6-question open-ended survey (Multimedia Appendix 1) was developed to elicit participants’ challenges and experiences in virtual research. The survey focused on communication and technological challenges from the perspectives of the participant and the interviewer and was developed through research team discussions, a thorough literature review, and consultation with observation notes (ONs). During the second stage, participant and interviewer responses to the open-ended survey and interview ONs informed the development of an 18-item checklist (Multimedia Appendix 1). The checklist covered preinterview challenges (eg, recruitment- and communication-related) and interview difficulties (eg, questionnaire-, communication-, and technology-related) for participants and interviewers. Both tools underwent pilot testing among a group of participants and revisions were integrated.

### Interview Procedures and Data Collection

Participants provided written informed consent through a digital consent form. They selected an interview date and received a confirmation email with study details and the HL questionnaire. Participants used a computer, tablet, or smartphone to view the questionnaire and provided responses to questions to the research assistant over the phone. Participants with no compatible device received a physical copy of the ethics protocol and questionnaire via courier. Telephone interviews lasted 40‐80 minutes. All participants were offered a CAD $30 (US $21) honorarium. After completing the questionnaire, participants were given the virtual research survey or checklist, with responses recorded verbatim. The research assistant took observational notes during the interviews for further data contextualization. All observational notes, interview recordings, and transcripts were deidentified and stored using password-protected files on institutional servers.

### Data Analysis

Initially, 3 members of the research team (NT, AM, and YSC) thoroughly reviewed the survey or checklist and corresponding ONs. While doing so, each team member independently created a list of challenges inductively. Meetings were then held to develop a coding guide in which conceptually similar codes were combined, infrequent codes were removed, and definitions were developed.

The coding guide was then piloted with 2 coders (NT and AM), who separately coded a random 15% of the data with equal distribution of survey or checklist and initial or retest interviews. The goal was to achieve 80% interrater agreement during the pilot, a benchmark accepted by many researchers [37]. After the initial trial of coding, the desired agreement level was not reached. The coding framework was subsequently revised by NT and AM by once again removing unnecessary items, clarifying definitions, and combining similar items. The coders then coded a new, random 15% of the data (with equal distribution of survey or checklist and initial or retest interviews) and achieved the desired interrater agreement (≥80%).

Each coder then coded half of the remaining 85% of data. Subsequently, codes were reviewed by YSC, who identified incorrect or missed codes. The team then resolved issues through discussion. Any disagreements between the coder (either NT or AM) and the reviewer (YSC) were resolved by the other coder. Both direct quotes (DQs) and ONs were coded. Following coding, thematic analysis [38] took place. The codes from the framework were discussed and synthesized into larger overarching themes. Constant feedback on the emerging themes was elicited from team members. Themes not explicitly relating to virtual challenges were removed.

## Results

### Overview

From June to October 2020, participants completed a virtual phone interview where they completed the questionnaire with a research assistant. Some of these were considered initial interviews, where the participant filled out the questionnaire for the first time (n=185) and completed a postinterview checklist (n=36) or survey on virtual research (n=149). To test the HL tool’s reproducibility, 110 participants completed another interview, with the postinterview checklist (n=79) or survey (n=31), on their experience with virtual research challenges. Participants primarily identified as female (122/185, 65.95%), were older than 50 years (167/185, 90.27%), had asthma (108/185, 58.38%), and had a disease duration of less than 20 years (110/185, 59.46%). Additional participant characteristics can be found in Table 1.

**Table 1.** Patient characteristics (N=185).

Characteristic	Initial interview (n=185)	Retest interview (n=110)
Form, n (%)		
Checklist	36 (19.46)	149 (80.54)
Survey	79 (71.82)	31 (28.18)
Self-reported sex, n (%)		
Female	122 (65.95)	63 (34.05)
Male	75 68.18)	35 (31.82)
Condition, n (%)		
Asthma	108 (58.38)	77 (41.62)
COPD^a^/ACO^b^	65 (59.09)	45 (40.91)
Age (years), range, n (%)		
<50	18 (9.73)	9 (8.18)
50-70	80 (43.24)	50 (45.45)
>70	87 (47.03)	51 (46.36)
Self-reported ethnicity, n (%)		
White	159 (85.95)	26 (14.05)
Non-White	92 (83.64)	18 (16.36)
Education level, n (%)		
High school diploma or less	55 (29.73)	130 (70.27)
Post high school diploma	29 (26.36)	81 (73.64)
Disease duration (years), n (%)		
<20	110 (59.46)	9 (53.64)
20-40	50 (27.03)	33 (30)
>40	24 (12.97)	17 (15.45)
Unreported	1 (0.54)	1 (0.91)
First language, n (%)		
English	160 (86.49)	25 (13.51)
Non-English	94 (85.45)	16 (14.55)
Received education for condition, n (%)		
No	47 (25.41)	28 (25.45)
Yes	137 (74.05)	81 (73.64)
Unreported	1 (0.54)	1 (0.91)
Primary education source, n (%)		
Family doctor	116 (62.70)	66 (60)
Multiple	8 (4.32)	3 (2.73)
Other	6 (3.24)	3 (2.73)
Pharmacist	3 (1.62)	2 (1.82)
Respiratory educator	5 (2.70)	3 (2.73)
Respirologist	47 (25.41)	33 (30)

a COPD: chronic obstructive pulmonary disease.

b ACO: asthma-COPD overlap.

The following sections describe the 6 overarching themes and 23 subthemes derived from analysis: theme 1: overall experience with virtual research, theme 2: clarity of instructions, theme 3: technology, theme 4: communication, theme 5: condition and cognition, and theme 6: logistics. Participants’ direct quotes are indicated by “DQ” and researchers’ observation notes with “ON.” Identifiers for each participant, denoted by the letters “HL” and followed by a number, are used to anonymously identify which interviews the quotes or notes came from. Textbox 1 contains additional DQs and ONs from the 6 themes.

Textbox 1. **Participants’ direct quotes and researchers’ observation notes.**

**Theme 1: overall experience with virtual research**

*Positive overall telehealth experience*

- Overall, he was satisfied with the questionnaire and thought that the questions are well made. “I had a very pleasant experience being interviewed today.” [HL01, ON]
- She found the questionnaire interesting, learnt a few things, and [it] enabled her to think about certain things with some of the questions that she normally would not have. [HL68, ON]
- Thought [that the] interview went very well and didn’t know what to expect. “I was able to provide information about me and was happy that I was able to participate and enjoyed being a part of the study. The study had a purpose.” [HL101, DQ]
- “I think it was very worthwhile accumulating more data...health professionals are learning more and more and they’re acquiring and making better results for patients.” [HL101, DQ]
- He said that the interview was very helpful as he had never thought [about] where and from whom [to] find the health information. [HL109, ON]
- Participant expressed [a] good experience. She felt her participation made her more aware, to ask more questions about her asthma which is a good thing. [HL123, ON]
- At the end, she said: “I’m glad to participate in the study. It’s helpful and I learned a lot.” [HL162, ON]

*Negative overall telehealth experience*

- For the last section of the questionnaire, he refused to answer three questions because he thought they were vague and [weren’t] worth answering. [HL21, ON]
- He mentioned that the questionnaire was very elementary and [at a] grade 6 or 7 level and complained that it was not helpful at all. [HL177, ON]

*Wants to be updated*

- At the end of the interview he stated that he would like to receive the results of this study. [HL24, ON]
- She also mentioned that if in the future we develop a tool with information for people with asthma and how to manage it she would like to receive it. [HL29, ON]
- He was happy about participating and contributing to the study and asked about the next steps. [HL45, ON]
- “I’m just wondering, is it possible to be notified of the study results after everything is complete?” [HL65, DQ]
- After completing the questionnaire, she asked what we will be doing with the data collected and said she would like to receive a copy of our paper after it is published. [HL85, ON]

**Theme 2: clarity of instructions**

*Documents*

- Clarity of instructions over the phone and email about the consent form and meeting receipt weren’t really clear from the beginning. She mentioned how she was confused about it to me right when we first talked as the consent form that was sent was not interactive. [HL18, ON]
- “There are lots of emails and I’m confused.” [HL178, DQ]

*Email*

- Wasn’t obvious that there was an attachment of the questionnaire on the email. [HL47, ON]
- “You should send the emails individually [rather] than grouping them together”—he didn’t appreciate the fact that there were three [e-form] emails sent in one setting. [He had to receive 3 [e-form] forms because he didn’t sign the meeting receipt from the first interview and had another meeting receipt form and the consent form sent doing the retest interview]. [HL69, ON]

*Interview*

- She also mentioned that she was not aware that the better way to do the interview was on a computer rather than an iPhone. [HL37, ON]
- He also said he had looked over the questionnaire but didn’t realize he needed to have it open in front of him. However, it only took a minute or two for him to find the email and open it. [HL179, ON]

*Purpose*

- Another comment he mentioned was that the literacy study that we are conducting is tricky because he doesn’t know whether our objective of this study [is] to test the individual’s capability to follow instructions or whether to test how much they know based [on] their own knowledge. [HL34, ON]
- “In [the] first interview, the purpose of the study was not clear, [and I even] received a call and e-mail from the study, but, I now have better understanding regarding the health literacy after the retest.” [HL116, DQ]
- When we got to the passages, she was wondering what the purpose of the passages were if they didn’t apply to her [e.g., she didn’t use inhalers]. [HL121, ON]
- “What is the purpose of the study? I don’t get if the study is trying to understand how well people can understand material or how accurate the measurement tool is.” [HL181, DQ]

*Recruitment*

- She wasn’t sure if she was eligible initially. [HL96, ON]
- Yes, I had to communicate with her over email multiple times because she was unsure of [1] if she was eligible; and [2] when she had to sign the receipt form. [HL96, ON]

**Theme 3: technology**

*Device and internet*

- [The] receipt form could not be signed through [her] iPhone as it would not let her complete the signature portion, [so] she had to switch to her iPad to complete it. [HL20, ON]
- His computer didn’t start. [HL23, ON]
- [The participant] viewed [the study documents] on [an] iPhone and had different things popping up [e.g., copy/highlight tool]. [HL62, ON]
- “Problems came from my computer. It was just downloading for a long time. Laptops can be slow at times.” [HL65, DQ]
- Participant may have an older computer that couldn’t open up all the graphics or text. [HL68, ON]
- His laptop has a scrolling issue so that was a bit frustrating for him at times. [HL139, ON]
- She needed to charge her computer in the middle of interview and said: “I got frustrated with my computer.” [HL187, ON]
- “Just my issue with my internet.” [HL104, DQ]
- “Why isn’t it letting me open it?”—[the participant] had trouble opening the questionnaire due to [a] slow internet connection. [HL104, ON]

*Knowledge*

- He was an older gentleman and needed help using his computer and email to sign the forms and open and view the questionnaire. [HL01, ON]
- She scrolled past questions on the questionnaire occasionally but said that this was because she doesn’t handle computers much and not because of the layout of the questionnaire. [HL15, ON]
- He had a woman who I assume was his caretaker to help him with the emails and technology-based aspects of this study, as he wouldn’t have been able to work the computer on his own. [HL49, ON]
- She said: I am not a computer person and signing a document on the computer is hard for me. [HL105, ON]
- I prefer a paper in front of me rather than computer and [an] in person interview is more convenient. [HL105, ON]
- “I’m not very computer savvy so when there are many things coming up on my computer it throws me off.” [HL157, DQ]
- “I am over seventy years old, this [working online] is foreign to me.” [HL157, DQ]

*Navigation*

- [The participant] had trouble scrolling [and] would skip pages by accident. [HL05, ON]
- “I don’t like scrolling through documents over email because I don’t have a mouse and it hurts my hand to scroll after a while, so now my hand hurts.” [HL12, DQ]
- She had trouble scrolling back and forth when doing the Air Quality passage questions: “I’m losing track because I keep going back and forth.” [HL65, ON]
- Would get lost when scrolling back and forth through the Weather and Air Quality section; he would have to ask me to tell him what question he was on. This happened three times during the interview. [HL69, ON]
- The constant scrolling up and down with the passages and answering the questions were somewhat cumbersome since she was doing it on her phone. She lost the page on several occasions. [HL86, ON]
- [The participant] had trouble scrolling and lost her place in the questionnaire many times. [HL128, ON]
- She lost the pages frequently while looking back the passages and said: “I’m scrolling the wrong way.” [HL134, ON]
- “Again, the scrolling back and forth was challenging. I lost track of which question I was answering several times having to go back and forth.” [HL168, DQ]

*Document opening*

- The participant struggled quite a bit getting the documents open. [HL43, ON]
- As the participant mentioned, she had trouble finding the email with the questionnaire attached and opening the attachments. [HL72, ON]
- “Bloody hell. Oh, Christmas tree! I just got rid of it! How did I do that?...What happened is, I touched something and I got rid...my thing went away. I have to open the document again.” [HL138, DQ]
- The email logged her out, so she had to re-open the document 3 times during the interview. [HL138, ON]

*Document retrieval*

- She needed help finding the study email in her inbox and opening the PDF file. [HL16, ON]
- Once he filled out the consent form, he was initially confused about where to find the questionnaire, as he thought that it was also on [the e-consent form website]; however, after clarifying it was in his email he required the document to be resent to him. [HL39, ON]
- The participant lost the initial email from [the principal investigator] at some point, so I had to send her another one with the questionnaire attached. [HL118, ON]
- [The patient did] not initially notice the email containing the link [for] signing the consent form. [The] patient was only looking at the email [that the principal investigator] sent. [HL125, ON]
- She hadn’t noticed [that] there was a questionnaire attached to her email. [HL150, ON]
- She asked me to wait until she could find the questionnaire. However, she called me back 24 minutes later. [HL186, ON]

*Consent form*

- The participant appeared to be slightly confused while carrying out the process of the e-signature; however, he was able to do it after further clarification. [HL01, ON]
- She had trouble signing and submitting the consent and meeting receipt forms because she wasn’t familiar with the process. [HL16, ON]
- She could not easily tell if she had successfully completed the [e-signature] forms. [HL20, ON]
- [The patient had] trouble signing the form online using the signing technology, had never done it before. [HL31, ON]
- Struggled with the informed consent form and providing an e-signature as she was using an iPad—however, she was able to figure it out in the end. [HL35, ON]
- She also had difficulty filling out her e-signature on her iPhone and ultimately had to move to computer. [HL37, ON]
- “I had problem signing the form. It wouldn’t take my signature, so I had to get my wife to do it for me.” [HL62, DQ]
- We had a few technical difficulties with the consent form as it was harder for her to go through it and sign it. We had to go through the steps slowly, and after around 15 minutes, we finally signed both the documents. [HL162, ON]
- “I had problems with the actual signing of the paper. But that was the only technical glitch.” [HL170, DQ]

**Theme 4: communication**

*Contact*

- Email following up the initial phone. [HL26, ON]
- “[I] sent an email back to [the principal investigator] but I didn’t hear back but I did receive a few reminders to complete the [online consent form].” [HL114, DQ]
- “I did leave a message with [the principal investigator] about the witness but I didn’t get a response and I figure I would clear it up with [the research assistant] when she called.” [HL168, DQ]

*Interruption*

- “Just going to get the dog out of this room. He is a puppy and likes to come after me and bark”—before the interview began she had to make sure she wouldn’t be distracted while doing the interview at home. [HL36, ON]
- He had to leave the interview for a second to talk to his family member. [HL69, ON]
- It was difficult to obtain his responses because there was a lot of static over the phone and we got disconnected a few times due to [the] phone running out of battery. [HL80, ON]
- Throughout the interview, the phone would cut out and I would not be able to hear some of her responses, so I had to repeat and clarify multiple times. [HL100, ON]
- [The participant]had to take a phone call in the middle of the interview. [HL124, ON]
- One of the handsets for the patient’s home phone [ran out of power] near the end of the interview, requiring that I call back. [HL125, ON]
- In the middle of [the] interview she said: “I have to take something out of my oven and I’ll be right back.” [HL132, ON]
- “I only had one problem. It was just the attention stuff going on around me.” She was referring to distractions, including her cat. [HL167, ON]
- She had a phone call in the middle of the interview. [HL169, ON]
- The participant’s dog was constantly barking in the background. She later had to leave the interview for about 10 seconds to take care of her dog. [HL170, ON]

*Interview*

- Could not hear the [participant] clearly during [the] initial call [as she] spoke very quickly. [HL04, ON]
- Her only issues were [the] quality of [the] call [couldn’t always understand or hear]. [HL40, ON]
- He went to find his medications to read and he was difficult to hear because he put it on speaker phone and read them from where he was. [HL67, ON]
- When we first started to conduct the interview, the participant mentioned she was not able to hear me that well, so I switched to speaker phone and spoke a bit louder, and that seemed to solve the issue. [HL89, ON]
- She mentioned that there were parts where I’ve cut out and she couldn’t hear me well. I told her to let me know if she’s experiencing difficulties in hearing me, and I’ll slow down and repeat what I’ve said. [HL172, ON]

**Theme 5: condition and cognition**

*Anxiety or worries*

- [The participant] was worried that he was taking too long to complete questions. [HL, 23, ON]
- She mentioned [that] she felt pressed for time and felt some pressure to read through the passage as quickly as possible. [HL35, ON]
- She did mention that she thought she would have answered better if she had been able to see the questionnaire before the phone interview, as seeing it for the first time while on the phone cause her some anxiety. [HL72, ON]
- She also commented she felt anxious on the mental process of having to read through each passage and having to provide a response. [HL73, ON]
- She mentioned that she felt pressure when having to respond over the phone to the research assistant. I assured her that she did a great job and there are no correct or incorrect answers. [HL110, ON]
- “Because you are inevitably required to read on one page and asked to answer questions on another page, it caused a little bit of mental distress.” [HL181, DQ]

*Font, format, and eyesight*

- “I’m not sure what that number is...it looks like 15? But there is no 15!” [HL02, DQ]
- “Is that E5 I see? I cannot really tell.” [HL02, DQ]
- “It was annoying because you guys have various fonts used in the document.” [HL12, DQ]
- [Asthma Control Test] section—“Excruciatingly small print!” [HL21, ON]
- When [completing] the Hospital Map section: “[The] numbers are so small, it is almost unreadable!”—he was very dissatisfied with the font size. [HL21, ON]
- “I can’t read the damn map!”—he complained about the small font size for the Hospital Map section. [HL47, ON]
- “I wish I could have both the question and the passage in the same page.” [HL105, DQ]
- “Yes, it says don’t print out the questionnaire which would [have] made it much easier for me if I had it in front of me...instead [of] having to scroll back and forth.” [HL106, DQ]
- “I’ve got a cataract in one eye so it makes it difficult to see the hospital map.” [HL111, DQ]
- “It would be better if the participants can see the passages and questions at the same time. People over 70 dealing with eye problems such as [a] cataract [may find it] difficult for them to scroll back and forward.” [HL134, DQ]
- “For old people, you should make it big enough so that it could be read.” [HL173, DQ]

*Condition*

- We were able to get through half of the questionnaire before she started to feel ill and we had to reschedule. [HL40, ON]
- The participant said she has a hearing aid which may have led to her not hearing some parts of the questionnaire. [HL98, ON]
- He indicated that he had difficulty [talking] loudly and for a long time. [HL139, ON]
- During the interview he frequently coughed and cleared his throat. [HL109, ON]
- She could not speak very well because of voice hoarseness and had to clear her throat every minute. [HL161, ON]
- She had her wrist broken and it was difficult for her to work on [a] computer with one hand. [HL161, ON]
- During the interview, I could hear her wheezing sounds over the phone. [HL166, ON]

**Theme 6: logistics**

*Confidentiality*

- While filling out the e-signature document she questioned whether the website was secure in terms of getting hacked. [HL12, ON]
- He had some questions at the end about the anonymity of results which I had some trouble answering as I’m not completely knowledgeable of the whole process. However, I referred him to [the principal investigator] for more information. [HL28, ON]
- She also had a question as to whether the phone call was being recorded, but I told her it wasn’t and that I was just recording on a computer the answers that she gave me to the questions. This seemed like a concern about privacy but my answer was OK with her. [HL99, ON]

*Rescheduling and delays*

- During the initial questionnaire interview call on Tuesday, the participant asked for a postponement to a later time in the same day. The second scheduled call was also unfruitful as she was still travelling back home. [HL40, ON]
- We were able to get through half of the questionnaire before she started to feel ill and we had to reschedule. [HL40, ON]
- She was unreachable for the first 17 minutes—she was walking her dog. [The] interview ended up starting 25 minutes late. [HL104, ON]
- Her cell phone was silent so the interview was delayed. [HL130, ON]
- She asked me to wait until she could find the questionnaire. However, she called me back 24 minutes later. [HL186, ON]

*Team error*

- There was a typo in the participant’s email, and she had not received the documents initially so the documents had to be forwarded to her. [HL22, ON]
- She said that with the initial compensation, she did not receive the password, and was not able to access it. [HL22, ON]
- The wrong number was listed in the [study contact list], [so] I had to look for the corresponding number using the call ID system that we created. This led to the interview being conducted 30 minutes late. [HL24, ON]
- Due to my impatience and my computer lagging, I accidentally was sending the participant multiple emails from the e-signature website. [HL148, ON]

### Theme 1: Overall Experience With Virtual Research

Participation was generally satisfying and meaningful to participants.

#### Overall Telehealth Experience

Most participants found their first experience with virtual research positive. They appreciated learning about relevant self-management topics through the questionnaire. This sentiment was captured by one individual who mentioned that it was both *“*educational and informative*”* [HL94, DQ].

#### Negative Overall Telehealth Experience

Few participants expressed dissatisfaction, with the main complaints surrounding the questionnaire, with one participant “[refusing] to answer three questions because he thought they were vague” [HL21, ON].

#### Wants to Be Updated

Participants took pride in contributing to research that might benefit others with similar health conditions and many expressed interest in learning the study’s outcomes. One asked “Is it possible to be notified of the study results after everything is complete?” [HL65, DQ]

### Theme 2: Clarity of Instructions

Instructions regarding the study’s requirements, delivered via email and phone, were sometimes a source of confusion.

#### Documents

For some participants, confusion arose from understanding which documents required signatures and the process of phone-based questionnaires. One asked “which [form] should be signed” [HL147, ON]. Additionally, some were unsure whether signed copies they received needed further action.

#### Email

Some participants struggled to discern the importance of the various emails they received. One participant thought that the content and documentation were excessive but assumed “it’s all necessary” [HL156, DQ].

#### Interview

Participants were occasionally caught off guard by the nature of the interview, especially those unfamiliar with the field of HL. One was confused over the method of data collection: “I thought I could fill out the questionnaire instead of verbally telling you my answers over the phone” [HL185, DQ]. The need for very clear expectations was expressed.

#### Purpose

Some participants questioned the purpose of the study and the tasks they were asked to perform, discussing the need for clarification and reiteration. One commented: *“*In [the] first interview, the purpose of the study was not clear” [HL116, DQ].

#### Recruitment

Despite eligibility screening during recruitment, some participants still had questions about their eligibility at the interview stage. For one participant, the research assistant noted: “although her doctor diagnosed her with COPD, she doesn’t have any symptoms and asked if she is still eligible to be part of this study” [HL175, ON].

### Theme 3: Technology

Virtual study conduction necessitated technological engagement, leading to several challenges.

#### Device and Internet

Participants sometimes grappled with device-related challenges. Issues included tablet or phone incompatibilities with document formats, unexpected system behaviors (eg, start-ups or restarts and low battery), and distractions from app notifications. For instance, one participant’s phone screen could not adequately display the full document: “the screen wasn’t big enough to have the full [file] format because I was opening the document from my cell phone” [HL62, DQ]. Poor internet signal caused difficulties in accessing documents and signing online forms.

#### Knowledge

For many, unfamiliarity with technology caused challenges opening documents, adjusting view settings, and signing digital forms. There was a preference among some for in-person interviews or alternatives such as Zoom: “It would have been easier if it was face-to-face or in-person...maybe even by Zoom” [HL157, DQ].

#### Navigation

Scrolling through the digital questionnaire was troublesome for some. In the digital format, participants often had to scroll to reference material to answer questions, leading to disorientation in the document. One lamented the constant movement or scrolling, feeling like they were going “up and down like a yo-yo” [HL16, ON].

#### Document Opening

A minority of participants encountered difficulties in opening study documents due to the absence of instructions on how to access PDF or Microsoft Word attachments within emails. For instance, a participant mentioned that they, “had trouble getting the questionnaire to open at first as an attachment to the email” [HL50, ON].

#### Document Retrieval

Some participants had difficulty retrieving study documents from emails misrouted to the “junk” folder or lost in their inbox. For example, “the emails had initially gone to her junk mail so there was a bit of difficulty on finding the documents” [HL37, ON].

#### Consent Form

Participants experienced various issues with the online consent form. Some reported that the provided link was nonfunctional: “the link to the consent form...did not work” [HL05, ON]. Mobile and tablet users faced navigation and compatibility issues, and the signature process was cumbersome for many, with one describing it as “doing etch-a-sketch with the mouse” [HL61, ON]. A frequent concern was the lack of submission confirmation, leading participants to often seek verification from the research assistant.

### Theme 4: Communication

Communication issues occurred between research staff and participants throughout the research process. These challenges are categorized as challenges with contacting the research team, interruptions during the interview, and other challenges.

#### Contact

On rare occasions, participants had trouble communicating with the research team postrecruitment and prequestionnaire or interview, which made scheduling difficult. One reported that they “didn’t know how to get a hold of [the research team] after [the] initial call” [HL26, ON].

#### Interruption

Telephone interviews, conducted at a location of participants’ convenience, led to interruptions from external sources, such as pets, family members, other calls, and phone connection issues. A notable instance was when a participant asked the researcher to hold the call for 6 minutes while answering another [HL08, ON].

#### Interview

Phone interviews sometimes had communication hitches from poor call quality, background disturbances, or participants’ swift responses. One research assistant reported: “[the] participant was reading out answers very quickly, I had to slow [them] down to keep up” [HL103, ON].

### Theme 5: Condition and Cognition

Some participants had health and/or cognitive conditions that caused difficulties during the research study.

#### Anxiety or Worries

Despite no set time limit, some participants indicated that they felt pressure to answer swiftly, as evidenced by one individual who felt the strain of “analyzing the passage while on the spot” [HL110, ON]. Another cause of stress for participants was feeling as though they were being assessed.

#### Font and Formatting (Eyesight)

Several participants found the font size in the questionnaire too small and often attributed this to their poor eyesight, as one mentioned: “numbers are too small” [HL16, ON].

#### Condition (Noneyesight)

Some participants had hearing difficulties which complicated the phone interview, while others had breathing issues due to their respiratory conditions. In the case of one participant, “[they] coughed a lot and asked for [a] 10-minute break in the middle of the interview and took [their] inhaler” [HL 154, ON].

### Theme 6: Logistics

There were also logistical and planning challenges that arose throughout the study.

#### Confidentiality

A few participants raised concerns about confidentiality, particularly regarding their initial contact (random callouts) and potential data sharing with third parties. Some individuals were also initially concerned with how their personal information might be used and how or where their information would be stored. One participant thought that “some of these studies share info with insurance and pharmaceutical companies [as] every year his list of allowed or covered medications [had] gone down” [HL138, ON].

#### Rescheduling and Delays

Interviews often faced rescheduling due to participants being unprepared, forgetful, falling ill, or having other commitments.

#### Team Error

Errors from the research team, although infrequent, did arise. Examples include incorrect email entries and honorarium transfer discrepancies, which were promptly addressed.

## Discussion

### Principal Findings

Our findings illustrate the experiences and challenges faced by participants in a virtual research study validating an online HL tool. Six themes emerged, showing that participants generally reported a positive experience but faced challenges with technology, mental fatigue, and data confidentiality concerns. Communication issues, such as distractions and unclear instructions, were also noted.

The virtual nature of this study allowed participants to engage at a convenient time and location, eliminating travel-related time and cost burdens. Individuals whose health limited their ability to attend in-person sessions were also able to participate, enhancing the diversity of the sample. These 2 advantages align with literature supporting virtual research as more cost-effective and time-efficient, allowing for broader geographical reach and greater sample diversity [4-6,8]. However, later we discuss how factors such as participant response times may not always make virtual research more time- and cost-effective than in-person research.

A common concern with virtual methods is the exclusion of individuals with lower technological skill, particularly older adults [39,40]. In this study, most participants were aged 50 years or older, with 43% (80/185) being aged 50‐70 years and 47% (87/185) being older than 70 years. Additionally, internet use among older adults in British Columbia is high, with 88% of adults aged 45-64 years and 56% of adults aged 65 years and older using the internet [41]. Despite high rates of internet use, the digital divide persists among older adults, particularly those with lower socioeconomic status, limited education, and rural residences [42-44]. Even for older adults with internet access, disparities exist in the breadth and depth of digital engagement, with many lacking the skills to perform health-related tasks such as accessing patient portals, booking appointments, or participating in telehealth [43-45]. Barriers extend beyond access to include physical and cognitive impairments, privacy concerns, lack of social support, and preference for face-to-face interactions [46-49]. Without interventions such as digital literacy training or infrastructure improvements, virtual methods can risk exacerbating health disparities by excluding older adults with the greatest health needs and fewest resources [44,50,51].

Challenges arose with communication about study expectations. Despite regular contact through email and phone, participants occasionally felt unclear about the study’s objectives, the data collection process, and documents requiring their signature before receiving further clarification. This highlights the need for clear, concise instructions and avoidance of overburdening participants, while also ensuring opportunities to ask questions. Research teams should create detailed and user-friendly instructional materials and provide multiple avenues for participants to seek clarification. These recommendations also apply to in-person research, where recruitment and interview coordination often also occur online [4].

Participation in virtual studies requires technology and internet access. In this study, participants used various devices including laptops, tablets, and smartphones. While this allowed a wide range of individuals to participate, technological skills varied, leading to challenges such as difficulty opening attachments, navigating documents, and phone users struggling to display documents in full. The research assistants supported these participants by asking them to explain the issue they were experiencing in their own words and then providing tangible steps at their technology literacy level. This contributed to varied amounts of time spent with each participant. Thus, virtual research may not always be more cost-effective or time-efficient than in-person research and depends on the individual participant [52]. Some virtual studies have reported additional time spent supporting participants with technology and higher participant cancellation and no-show rates [53]. This was also observed in our study as participants sometimes had to reschedule interviews due to being unprepared, forgetting, falling sick, or having multiple commitments at the same time. While rescheduling may happen more often in virtual studies, it can be seen as an advantage as the virtual environment allows for more flexible rescheduling instead of participant dropouts [4,7].

Throughout the study, communication issues were encountered. Since virtual studies can be conducted from any location, there were interruptions from external sources such as family members, pets, other calls, and connection issues. These interruptions can be detrimental to the quality of data collected, as distractions can lead to less contextually rich information provided, and poor call quality can result in missing information. Additionally, some participants also experienced mental fatigue and difficulty focusing, further contributing to decreased data quality [20]. These challenges are common in virtual research [5,18,52,54]. To avoid this, researchers can proactively ask participants to prepare a quiet environment to minimize distractions, ensure that questionnaires and interviews are not too lengthy, take breaks when necessary, and offer adequate incentives to maintain participant engagement.

This study generated several practical lessons for future virtual research design. In summary, participants benefited from simplified and consolidated communication. Sending fewer emails with clearly labeled attachments and step-by-step instructions can help reduce confusion. Electronic consent procedures should also be optimized for mobile devices and include confirmation messages indicating successful submission. Document design should minimize scrolling demands and allow participants to view passages and questions simultaneously where possible, particularly for older adults or those with visual impairments. Researchers should anticipate varying levels of digital literacy and allocate sufficient time for individualized technical support. Finally, participants should be encouraged to prepare a quiet environment and be informed in advance about interview expectations, duration, and required materials to minimize interruptions and anxiety during participation.

Documenting patient experiences in virtual research is fundamental to participatory medicine and patient-centered research. Participatory medicine is guided by the phrase “nothing about me without me” and incorporates patient perspectives at every step of the research process, from question selection and study design through data collection, analysis, and dissemination [55-58]. Participants in this study emphasized the importance of clear communication, opportunities for clarification, technical support, and understanding the purpose of research. Many participants also expressed a desire to receive study findings and remain informed about how their contributions would be used. These observations reinforce the principle that participants should be treated as active partners in research. By capturing the lived experiences of patients with CRD with virtual research methods, this study contributes practical insights for designing patient-centered research.

### Strengths and Limitations

This study has several strengths. The virtual nature allowed for broader participation, likely leading to a more representative sample. Furthermore, the data analysis used investigator triangulation to maintain consistency between coders. However, limitations were identified, including potential sampling bias by excluding participants without access to reliable technology. To mitigate this, we mailed surveys to a couple of participants who requested it. Furthermore, inconsistent time was spent with each participant due to the varied technological skill level. However, this variety meant that perspectives were being collected from a diverse set of participants and did not favor participants with better technological skill. Future research should focus on comparing synchronous and asynchronous methods and explore participant engagement strategies. Additionally, addressing ethical considerations will help optimize the effectiveness of virtual research.

In conclusion, this study identified the experiences and challenges of patients with CRD with virtual research. Participants generally reported a positive experience and expressed satisfaction to participate in research that may benefit individuals with similar medical conditions. Challenges mainly encompassed technological barriers, the need for clear and concise instructions, mental fatigue, and concerns about data confidentiality. These findings emphasize the importance of providing comprehensive technical support, clear and concise instructions, and assurance of secure data storage methods to ensure effective, ethical, and inclusive virtual research. With these considerations, virtual research can remain a viable and valuable method, even among older adults. Future research should focus on enhancing participant engagement and improving digital technology accessibility post COVID-19 pandemic.

## Supplementary material

10.2196/80216 Multimedia Appendix 1 Sample survey and checklist.

## Abbreviations:

CRD: chronic respiratory disease
DQ: direct quote
HL: health literacy
ON: observer note
