# Supplementary material for: Experiences and Challenges of Patients With Chronic Respiratory Disease During a Virtual Research Study: Qualitative Study
Source: J Particip Med. 2026 Jul 9;18:e80216. doi: 10.2196/80216 (PMC13348803; doi:10.2196/80216)
Supplement: Multimedia Appendix 1 [file jopm-v18-e80216-s001.docx]

**Multimedia Appendix 1**

**SAMPLE OPEN-ENDED SURVEY**

Did you have any challenges with the clarity of instructions provided over the phone or by email? If so, what were they?

*If they mention challenges:* What might you change to reduce this challenge (ask about each challenge mentioned)?

Did you have any technical or technology-based challenges? If so, what were they?

*If they mention challenges:* What might you change to reduce this challenge (ask about each challenge mentioned)?

Did you have any other challenges that you haven't mentioned? If so, what were they?

*If they mention challenges:* What might you change to reduce this challenge (ask about each challenge mentioned)?

**Interviewer (fill out on own time)**

Did the participant have any challenges with the clarity of instructions provided over the phone or by email? If so, what were they?

Did the participant have any technical or technology-based challenges? If so, what were they?

Did the participant have any other challenges that you haven't mentioned? If so, what were they?

Did you, the interviewer, have any challenges? If so, what were they?

**SAMPLE CHECKLIST**

***Script (Complete before starting interview):*** Thank you for being a part of our study. Just before we begin the questionnaire, I’d like to ask you a few brief questions about your experience with the study so far, as we are assessing challenges with research conducted at a distance, both over the phone and computer. I’d like to know, did you have any challenge with:

| **Section 1: Pre-interview (Complete before starting interview)** | | | | |
| --- | --- | --- | --- | --- |
| Challenge | Participant | Participant Quote(s)  *If they had the challenge, ask them to please elaborate and how they might fix this challenge* | Interviewer (fill out after call) | Interviewer Notes (fill out after call) |
| **Recruitment** | | | | |
| Understanding your role in study |  |  |  |  |
| Understanding the study process provided verbally |  |  |  |  |
| Having any questions that weren't answered |  |  |  |  |
| **Communication** | | | | |
| Understanding and signing the consent form |  |  |  |  |
| The clarity of instructions in any emails |  |  |  |  |
| Reaching staff if needed before the time of the interview |  |  |  |  |
| **Other (fill in challenge - e.g. had to re-book, cancel interview)** | | | | |
|  |  |  |  |  |

***Script (Complete after interview):*** Thank you for participating in our study. Just before we finish, I’d like to ask you a few questions about your experience with the study as we are assessing challenges with tele-health studies, such as this study conducted with you. I’d like to know, did you have any challenge with:

| **Section 2: Interview (Complete after interview)** | | | | |
| --- | --- | --- | --- | --- |
| Challenge | Participant | Participant Quote(s)  *If they had the challenge, ask them to please elaborate and how they might fix this challenge* | Interviewer (fill out after call) | Interviewer Notes (fill out after call) |
| **Technology** | | |  |  |
| Opening study documents received by email |  |  |  |  |
| Retrieving study materials electronically |  |  |  |  |
| **Questionnaire** | | | | |
| Understanding passages/scenarios or have any formatting concerns |  |  |  |  |
| Understanding questions (e.g. answer based on information in passage, wording) |  |  |  |  |
| Viewing passage and questions (e.g. scrolling) |  |  |  |  |
| The length of the questionnaire (e.g. attention span) |  |  |  |  |
| **Communication difficulties** | | | | |
| Hearing me over the phone |  |  |  |  |
| Speaking pace |  |  |  |  |
| Understanding instructions for each section |  |  |  |  |
| My tone/accent, if it affected your answers at all (e.g. think you were wrong/right) |  |  |  |  |
| **Other (fill in challenge - e.g. interruptions, talking with others)** | | | | |
|  |  |  |  |  |

**CODING GUIDE**

**Summary of Codes**

|  | **Category** | **Description** |
| --- | --- | --- |
| **1. Overall Experience with Virtual Research** | **Positive Overall Experience** | Participant felt that the interview process over the phone did go smoothly [e.g., learned from the process, enjoyed speaking to someone, enjoyed benefiting future research, enjoyed helping people similar to themselves] |
|  | **Negative Overall Experience** | Participant felt that the interview process over the phone did not go smoothly - general comments, not categorized under a challenge code |
|  | **Wishes to be updated** | Would like to know results of the study |

| **2. Clarity of Instructions** | **Documents** | Understanding how/when to use certain study documents |
| --- | --- | --- |
|  | **Email** | Understanding the necessity of study emails and the information contained within them |
|  | **Interview** | Miscommunications regarding interview expectations [e.g., responding to questions over-the-phone]. NOT LENGTH OF QUESTIONNAIRE |
|  | **Purpose** | Understanding the purpose of the study [not expectations for interview] and/or their role in it |
|  | **Recruitment** | Miscommunications or unclear information shared during recruitment call[s], including questions about eligibility |

| **3. Technology** | **Device & Internet** | Device-related issues preventing a seamless process [e.g., device too old to open study documents, low device battery, iPad/tablet] Problems caused by unstable internet connection |
| --- | --- | --- |
|  | **Knowledge** | Non-consent form-related problems caused primarily due to a lack of technological knowledge/capabilities |
|  | **Navigation** | Issues with using technology to navigate the questionnaire |
|  | **Document Opening** | Issues with opening documents not due to technology [e.g., participant struggling with tech] |
|  | **Document Retrieval** | Trouble retrieving study documents [e.g., lost email] |
|  | **Consent Form** | Difficulty understanding how to sign/submit the virtual consent form [following instructions]; Difficulty using the technology required to sign/submit the virtual consent form [e.g., drawing signature with mouse]; OR other consent form issues OTHER THAN third party [e.g., SignX malfunctioning] |

| **4. Communication** | **Contact** | Trouble reaching team members |
| --- | --- | --- |
|  | **Interruption** | Interruption[s] during the interview [e.g., family member, pet] |
|  | **Interview** | Communication issues during the interview [e.g., speaking pace or tone] |

| **5. Condition and Cognition** | **Anxiety or Worries** | Any mention of anxiety, pressure, or unease experienced by the participant, including feeling rushed or being worried about the study process |
| --- | --- | --- |
|  | **Font and Formatting [Eyesight]** | Issues with the font/diagram used in the questionnaire [e.g., too small to read], including mention of eyes/eyesight being problematic |
|  | **Condition [non-eyesight]** | Difficulties during the research process stemming primarily from medical conditions [e.g., difficulty breathing while speaking], NOT including eyesight |

| **6. Logistics** | **Confidentiality** | Concerns over confidentiality, including how they were recruited |
| --- | --- | --- |
|  | **Rescheduling & Delays** | Delays or reschedules due to the participant not being available for the agreed upon interview time |
|  | **Team Error** | Problems attributable primarily to the research team [e.g., forgetting to send/attach study documents] |
